# MACC1 induces metastasis in ovarian carcinoma by upregulating hepatocyte growth factor receptor c-MET

**DOI:** 10.3892/ol.2014.2184

**Published:** 2014-05-27

**Authors:** XIU-JIE SHENG, ZHEN LI, MAN SUN, ZHI-HUI WANG, DONG-MEI ZHOU, JIAN-QI LI, QIN ZHAO, XIAO-FANG SUN, QI-CAI LIU

**Affiliations:** 1 Department of Obstetrics and Gynecology, The Third Affiliated Hospital of Guangzhou Medical University, Guangzhou, Guangdong 510150, P.R. China; 2 Key Laboratory of Reproduction and Genetics of Guangdong Higher Education Institutes, Guangzhou, Guangdong 510150, P.R. China; 3 Experimental Medical Research Center of Guangzhou Medical University, Guangzhou, Guangdong 510182, P.R. China

**Keywords:** metastasis-associated in colon cancer 1, metastasis, ovarian carcinoma, c-MET

## Abstract

Metastasis-associated in colon cancer 1 (MACC1) is a newly identified gene that has been shown to promote tumor cell invasion and metastasis. The present study investigated the effect of MACC1 downregulation on the biological characteristics of the ovarian cancer OVCAR3 cell line. In this study, MACC1 expression was blocked using the RNA interference technique. The downregulation of MACC1 mRNA and protein expression was confirmed using quantitative polymerase chain reaction and western blot analysis. The proliferative activity and adhesion rate of the cells were detected using cell counting kit-8 and a cell adhesion assay, while cell invasion was determined using a Matrigel invasion assay and migration capacity was observed using migration and wound-healing assays. A tube formation assay was also used to examine the angiogenic capacity of cells, and a luciferase assay was performed to assess whether MACC1 binds to the c-MET gene. The MACC1 mRNA and protein expression levels were significantly downregulated using sequence-specific small interfering RNA (siRNA). The inhibition of MACC1 expression markedly decreased the invasive, metastatic and angiogenic capacities of the cells, but only slightly inhibited growth and adhesion. In addition, a putative MACC1-binding site was identified in the 3′-untranslated region of c-MET. MACC1-siRNA was also found to significantly reduce the expression of the c-MET protein and a luciferase reporter assay confirmed that c-MET was the target gene of MACC1. These results demonstrated that the attenuation of MACC1 suppresses cell invasion and migration and that MACC1 may regulate cell metastasis through targeting the expression of c-MET. Inhibition of the function of MACC1 may represent a new strategy for treating ovarian cancer.

## Introduction

Ovarian cancer is the fifth leading cause of cancer-related mortality in females in the USA. Although substantial improvements have been made in ovarian cancer research, the five-year survival rate remains extremely low and the overall cure rate remains poor. Metastasis continues to be a major clinical challenge in the treatment of ovarian cancer (1,2), and an improved understanding of the molecular mechanisms underlying ovarian cancer invasion and metastasis may lead to the development of more effective therapeutic strategies. Metastasis is regulated by a number of factors and diverse mechanisms. Metastasis-associated in colon cancer 1 (MACC1) (3) was initially shown to promote the metastatic capacities of colorectal cancer, and clinical studies have indicated that it may be an independent prognostic indicator of recurrence and disease-free survival (DFS). Further studies have shown that MACC1 is overexpressed not only in colon cancer (4), but also in other carcinomas, including hepatic and lung cancer (5,6). Various studies have also revealed that the hepatocyte growth factor (HGF)/c-MET signaling pathway is key in carcinogenesis (7,8). Stein *et al* (3,9) demonstrated that MACC1-induced tumorigenesis correlates with HGF/c-MET signaling. MACC1 is a transcription factor that binds to the promoter of c-MET to stimulate its transcription, ultimately leading to the activation of the HGF/c-MET signaling pathway. In the present study, a MACC1-specific small interfering RNA (siRNA) was constructed, and its effects on adhesion, proliferation, migration, invasion and angiogenesis were assessed. Finally, a luciferase reporter assay and western blot analysis were used to confirm whether MACC1 functions as a metastatic promoter in ovarian cancer by targeting c-MET.

## Materials and methods

### Cell culture and siRNA transfection

The human ovarian cancer OVCAR3 cell line was purchased from the American Type Culture Collection (Manassas, VA, USA) and grown in Dulbecco’s modified Eagle’s medium (DMEM; Gibco-BRL, Carlsbad, CA, USA) supplemented with 10% fetal bovine serum (FBS; Gibco-BRL) and antibiotics (100 U/ml penicillin and 100 μg/ml streptomycin) at 37°C in a humidified incubator containing 5% CO_2_. Human umbilical venous endothelial cells (HUVECs) were obtained from the Institute of Biochemistry and Cell Biology of the Chinese Academy of Science (GenePharma Co., Shanghai, China) and cultured in Kaighn’s modified Ham’s F-12K medium (Mediatech, Inc., Manassas, VA, USA) supplemented with endothelial cell growth supplement (BD Biosciences, Mississauga, ON, Canada) and 10% FBS. MACC1-siRNA or a non-specific siRNA (Shanghai, China) was transfected into cells using Lipofectamine 2000 (Invitrogen Life Technologies, Carlsbad, CA, USA) according to the manufacturer’s instructions. Based on design principles and the MACC1 mRNA sequence, three siRNA sequences that targeted MACC1 and one siRNA sequence for use as a negative control were designed. The first sequence used to target MACC1 was 5′-GCCACCAUUUGGGAUUAUATT-3′, the second sequence was 5′-CACCCUUCGUGGUAAUAAUTT-3′ and the third sequence was 5′-GCCCGUUGUUGGAAAUCAUTT-3′. The negative control sequence used was 5′-UUCUCCGAACGUGUCACGUTT-3′.

### Quantitative polymerase chain reaction (qPCR)

Total RNA was extracted from the cells using TRIzol reagent (Takara Bio, Inc., Shiga, Japan) and reverse-transcribed into cDNA using the Prime Script RT reagent kit (Takara Bio, Inc.) according to the manufacturer’s instructions. The RNA was then analyzed by qPCR using SYBR Premix Ex Taq™ (Takara Bio, Inc.). The sequences of the primers used were as follows: MACC1 forward, 5′-GGCATTGTCCTGGTGTGGT-3′ and reverse, 5′-CACTCCTTCACCCCTGCTATCT-3′; and GAPDH forward, 5′-GCACCGTCAAGGCTGAGAAC-3′ and reverse, 5′-TGGTGAAGACGCCAGTGGA-3′. The GAPDH gene was used as an internal control for standardization in triplicate. The PCR conditions were as follows: 95°C for 30 sec; 40 cycles of 95°C for 5 sec, 60°C for 20 sec and 95°C for 15 sec; and 60°C for 1 min. PCR amplification was performed using the Mx3000P qPCR System (Stratagene California, La Jolla, CA, USA) and the comparative Ct (ΔΔCT) method was used to determine the fold change in expression.

### Western blot analysis

The cells were lysed in radioimmunoprecipitation buffer, and protein quantification was performed using the bicinchoninic acid assay (Sigma-Aldrich, St. Louis, MO, USA). A total of 30 μg of protein was separated using electrophoresis with a 12% SDS-PAGE gel. Following electrophoresis, the proteins were transferred to polyvinylidene fluoride membranes (Millipore, Billerica, MA, USA). Subsequent to being washed, the membranes were blocked with 5% skimmed milk for 1 h at 4°C and sequentially incubated with the following primary antibodies at the manufacturer’s recommended dilutions: rabbit antihuman polyclonal MACC1 (1:1,000; Abcam, Cambridge, MA, USA), c-MET (1:100; BioWorld Products, Inc., Visalia, CA, USA) and GAPDH (1:500; BioWorld Products, Inc.). The mixtures were then incubated overnight at 4°C on a rocking platform, followed by incubation with horseradish peroxidase-conjugated secondary antibodies. The proteins were detected using enhanced chemiluminescence plus detection reagents (Amersham Pharmacia Biotech, Tokyo, Japan) according to the manufacturer’s instructions. GAPDH was used as an endogenous protein for normalization.

### Cell growth assay

The cells were grown in 96-well culture plates, treated as indicated and cultured for 24, 48, 72 and 96 h. Next, the cells in each well containing 100 μl medium were incubated with 10 μl cell counting kit-8 (CCK-8; Beyotime Institute of Biotechnology, Shanghai, China) at 37°C for 2 h. The optical density (OD) of each well was then measured at 450 nm using a microplate reader (Thermo Fisher Scientific, Waltham, MA, USA).

### Cell adhesion assay

For the cell adhesion assay, 96-well plates were precoated with 50 μl BD Matrigel™ matrix (40 μg/ml; BD Biosciences, Heidelberg, Germany) at 4°C overnight. Prior to cell seeding, the wells were washed with phosphate-buffered saline (PBS) twice and blocked with 1% bovine serum albumin for 1 h at 37°C to prevent non-specific binding. The cells (100 μl) were trypsinized and seeded at a density of 1×10^4^ cells per coated well, incubated at 37°C for 60 or 90 min and then rinsed three times with PBS to remove the unattached cells. Fresh medium (100 μl) containing CCK-8 reagent (10 μl) was added to each well and the plates were incubated for an additional 2 h. The OD was then measured at 450 nm using a microplate reader. The OD values were proportional to the number of adherent cells; five duplicate wells were set up for each group.

### Wound-healing assay

The cells were grown to 95% confluence in DMEM containing 10% FBS in 24-well plates. A straight ‘wound’ was gently made by scratching the cells with a plastic pipette tip. The cells were washed twice with PBS and the dead cells were removed. Following culture in FBS-free medium for 0 and 24 h, wound healing was observed and images were captured using inverted phase contrast microscopy (CKX41, Olympus Corporation, Tokyo, Japan). The width of the scratch at the same position for 0 and 24 h was measured using Image Pro-Plus software (Media Cybernetics, Inc., Rockville, MD, USA).

### Cell migration and invasion assays

Tumor cell migration and invasion were assessed using a Transwell insert (8 μm; Corning, Inc., Corning, NY, USA). The OVCAR3 cells were grown to ~80% confluence and subsequently transfected with 120 nM MACC1-siRNA or a negative control. Subsequent to 24 h, the cells were harvested and washed with PBS. The cells (4×10^4^) were then resuspended in 200 *μ*l serum-free medium and seeded into the upper chamber of a Transwell insert. A total of 600 *μ*l DMEM containing 10% FBS as a chemoattractant was added to the lower chamber. For the invasion assay, the inserts were precoated with 30 *μ*l Matrigel and 5×10^4^ cells were added to the upper chamber. Following incubation at 37°C in a humidified atmosphere of 5% CO_2_ for 24 h, non-migrating (non-invading) cells were removed from the upper surface of the filter with a cotton-tipped swab. The cells on the lower surface of the filter were fixed in 4% paraformaldehyde and stained using crystal violet staining solution. Five random fields were counted at ×100 magnification. All the data that are presented are from at least three independent experiments that were performed in duplicate.

### In vitro tube formation assay

*In vitro* angiogenesis assays were performed using HUVECs plated on Matrigel. The OVCAR3 cells were cultured and treated in six-well plates containing fresh complete medium for 48 h, and 1 ml of conditioned medium was collected. The day prior to the tube formation assay, the BD Matrigel matrix was incubated overnight on ice. For the tube formation assay, 48-well plates were coated with Matrigel (100 μl per well) and allowed to polymerize at 37°C in a humidified atmosphere of 5% CO_2_ for 1 h. Next, 5×10^4^ HUVECs were suspended in 500 μl conditioned medium and added to the precoated 48-well plates. Following incubation for an additional 24 h, images were captured using an inverted phase contrast microscope and the tubular structures that had formed in the Matrigel were quantified by counting the number of connected cells in five random fields.

### Luciferase reporter assays

Luciferase reporter vectors were constructed as previously reported (10). Briefly, the pGL3-Basic vector (Promega Corporation, Madison, WI, USA) was used to generate luciferase reporter constructs. The 3′-untranslated region (UTR) of human c-MET was amplified from human genomic DNA using the following primers: 5′-CGGGGTACCCAGACTGCCTGAGCTGGGGGA-3′ (sense) and 5′-CCCAAGCTTGCGACCAGACTGAGGCGCTC-3′ (antisense). The amplicons were inserted into the *Kpn*I-*Hin*dIII restriction sites in the 3′-UTR of the hRluc gene in the pGL3-Basic vector. The constructs were confirmed by DNA sequencing and restriction enzyme digestion. The recombinant plasmid used was the pGL3-c-MET-promoter. In each well of a 48-well plate, 120 nM siRNA-MACC1 or a negative control was cotransfected with 0.3 μg of the firefly luciferase reporter vector and 0.01 μg pRL-SV40, using Lipofectamine 2000. Each transfection was performed in three separate wells. Luciferase assays were performed 24 h after transfection using the Dual Luciferase Reporter Assay System (Promega Corporation). Renilla luciferase activity was used to normalize firefly luciferase activity. Experiments were performed with each construct in triplicate.

### Statistical analysis

Quantitative data are presented as the mean ± standard deviation. All statistical analyses were performed with a one-way analysis of variance using SPSS version 17.0 (SPSS, Inc., Chicago, IL, USA). All experiments were performed at least in triplicate. P<0.05 was considered to indicate a statistically significant difference.

## Results

### Specific inhibition of MACC1 expression by MACC1-siRNA

**Figure 1:**
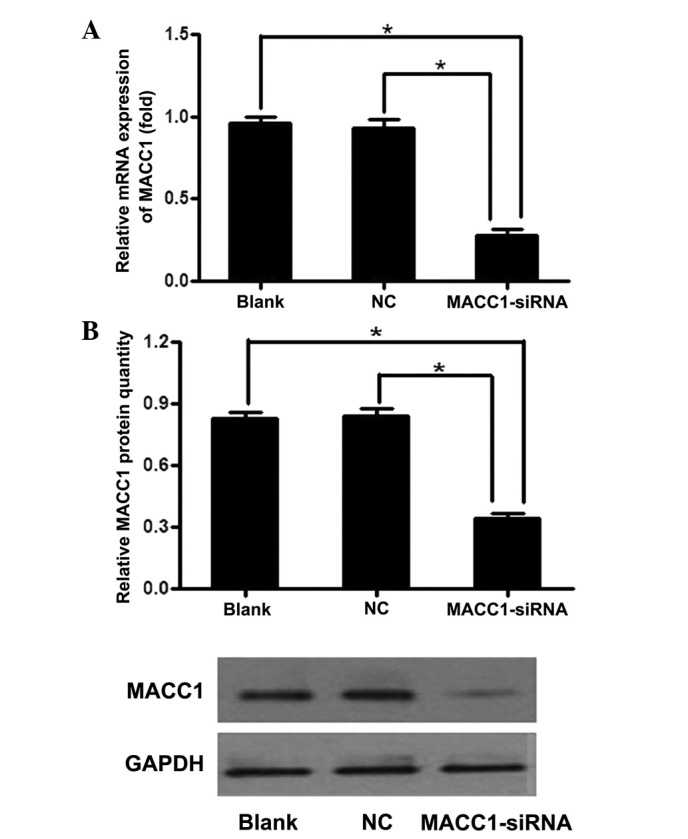
Knockdown of MACC1 expression by MACC1-siRNA in ovarian cancer. (A) The relative expression of MACC1 mRNA was analyzed using quantitative polymerase chain reaction (qPCR). (B) MACC1 protein expression was measured by western blot analysis. Data shown are presented as the mean ± standard deviation of a representative experiment that was performed in triplicate. ^*^P<0.05. MACC1, metastasis-associated in colon cancer 1; NC, negative control; siRNA, small interfering RNA.

The qPCR results confirmed that the first siRNA sequence was the most efficient at inhibiting the expression of MACC1. As shown by qPCR, the expression of MACC1 mRNA was significantly lower following transfection with MACC1-siRNA at 48 h, with an average inhibition of 75.7% compared with the control groups. Subsequently, the expression levels decreased (Fig. 1A). MACC1 protein expression was also decreased, with an average inhibition of 55.3% in the MACC1-siRNA group (Fig. 1B). These results indicated that MACC1-siRNA effectively suppresses MACC1 expression at the mRNA and protein levels in cells.

### MACC1 silencing inhibits ovarian cancer cell proliferation and adhesion

**Figure 2:**
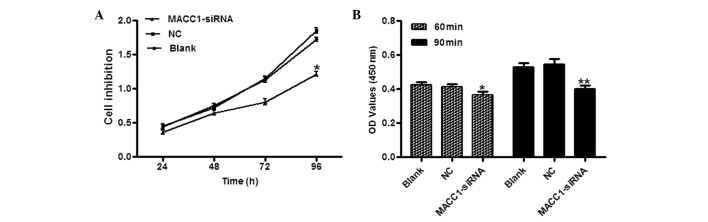
Effect of MACC1 on ovarian cancer cell proliferation and adhesion. (A) Knockdown of MACC1 inhibited ovarian cancer cell growth in a time-dependent manner compared with the controls. (B) Adhesion was examined by determining the OD at 450nm. MACC1 downregulation decreased the level of adhesion. ^*^P<0.05 vs. control and ^**^P<0.01 vs. control. MACC1, metastasis-associated in colon cancer 1; OD, optical density; NC, negative control; siRNA, small interfering RNA.

To assess the potential effect of MACC1 downregulation on proliferation and adhesion, cell adhesion and CCK-8 assays were employed. The results indicated that the MACC1-siRNA cells showed a time-dependent reduction in cell proliferation. Compared with the control groups, the inhibition rates were 10.2, 12.5, 24.0 and 31.7% at 24, 48, 72 and 96 h post-gene transfection, respectively (Fig. 2A). In the adhesion assay, the MACC1-siRNA cells exhibited reduced adhesion to the Matrigel matrix. The adhesion of the MACC1-siRNA-transfected cells was reduced by 14.3 and 25.9% at 60 and 90 min, respectively, compared with the control groups (Fig. 2B).

### MACC1 silencing suppresses ovarian cancer cell migration and invasion in vitro

**Figure 3:**
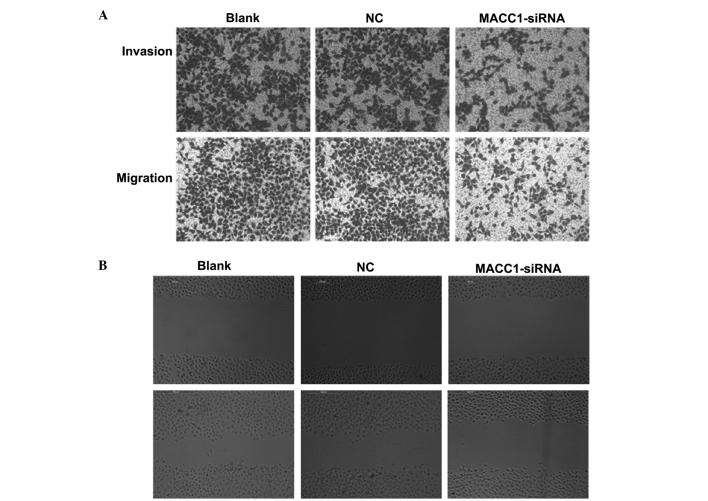
Inhibition of MACC1 attenuates the migration and invasion of OVCAR3 cells. (A) Cell migration and invasion were observed using Transwell chambers. The cells were fixed with cold methanol and then stained with crystal violet (magnification, ×100). (B) Wound-healing assay. Images were captured at 0 and 24 h. The migration capacity was significantly decreased in the MACC1-siRNA-transfected group compared with the control groups (magnification, ×100). MACC1, metastasis-associated in colon cancer 1; NC, negative control; siRNA, small interfering RNA.

Following MACC1-knockdown using transient transfection, the Transwell assay results showed that MACC1-knockdown resulted in a 51.0% reduction in cell migration and a 55.5% reduction in cell invasion compared with the control groups (Fig. 3A). In addition, the wound-healing assay indicated that MACC1-knockdown slowed the closure of the wounds, whereas the wounds healed more rapidly in the control groups (Fig. 3B). Taken together, these results indicated that MACC1 overexpression promotes the metastasis and invasion of ovarian cancer cells *in vitro*.

### Effect of MACC1-siRNA transfection on angiogenesis

**Figure 4:**
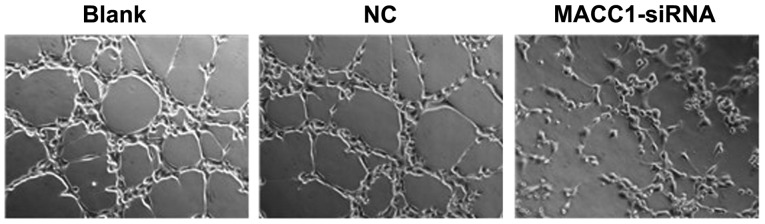
MACC1 suppression inhibits the *in vitro* angiogenic capabilities of ovarian cancer cells. Capillary-like tubes were observed and images were captured. Silencing MACC1 inhibited human umbilical venous endothelial cell (HUVEC) tube formation. The number of tubes formed per field was counted in five random fields for the blank control, NC and MACC1-knockdown groups (magnification, ×100). Data are presented as the mean ± standard deviation from three independent experiments. MACC1, metastasis-associated in colon cancer 1; NC, negative control; siRNA, small interfering RNA.

A tube formation assay using HUVECs was employed to determine the effects of MACC1 on ovarian cancer cell angiogenesis. As shown in Fig. 4, following treatment with the different supernatants for 24 h, extensive HUVEC tube formation was observed in the corresponding controls. However, when the HUVECs were treated with conditioned media from the MACC1-siRNA-transfected cells, the average number of complete tubular structures decreased by 39.5%. These results indicated that MACC1 inhibition markedly reduces the angiogenic capacity of the ovarian cancer cells.

### MACC1 activates the c-MET promoter and upregulates c-MET expression

**Figure 5:**
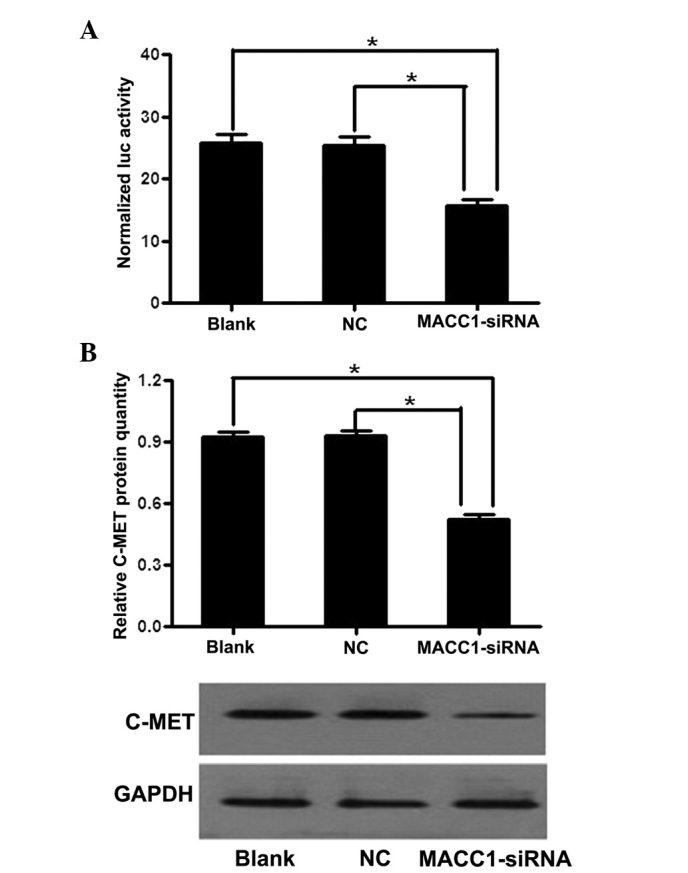
Effect of MACC1 on c-MET. (A) Luciferase activity of c-MET. OVCAR3 cells were co-transfected with a recombinant plasmid and MACC1-siRNA or a negative control. Luciferase assays were performed 24 h following gene transfection. The relative luciferase activity was defined as the luciferase value, normalized to Renilla levels. (B) Protein was extracted from the MACC1 silencing group and control groups, and c-MET protein levels were measured by western blot analysis. GAPDH was used as a loading control. ^*^P<0.05. MACC1, metastasis-associated in colon cancer 1; NC, negative control; siRNA, small interfering RNA; luc, luciferase.

Based on the evidence that c-MET is a target gene of MACC1 in colorectal cancer, the c-MET promoter fragments between −223 and +60 were amplified and cloned into a pGL3-basic vector, which is a positive regulatory element. Luciferase activity was analyzed by transiently transfecting the luciferase reporter cassette (pGL3-c-MET-promoter) into the OVCAR3 cells with or without MACC1-siRNA. In comparison with the control groups, transcriptional activity was markedly decreased in the cells that had been transfected with MACC1-siRNA, with an inhibition rate of 42.9% (Fig. 5A). Furthermore, MACC1-siRNA was found to significantly reduce c-MET protein expression levels to 41.5% of those observed in the control groups (Fig. 5B). These results indicated that MACC1 may regulate c-MET by activating its promoter between −223 and +60.

## Discussion

Tumor metastasis is characterized by a number of processes (11). At present, the primary cause of mortality in patients with solid cancer is tumor invasion and metastasis, however, the associated mechanisms remain unknown. The identification of biomarkers that can be used to monitor tumor invasion and metastasis in clinical practice may aid clinicians in effectively controlling tumor metastasis, determining the risk of recurrence and predicting patient survival (12).

MACC1, a recently identified metastasis-related gene, was identified by differential display qPCR of the normal colon, primary colon cancer and metastatic tissues (3). MACC1 is located on chromosome 7p21.1 and consists of 2,559 nucleotides encoding a protein containing 852 amino acids. MACC1 functions as a key activator of the HGF/c-MET signaling pathway, promoting colon cancer cell proliferation, invasion and metastasis in culture, and the growth and metastasis of tumors in xenograft models. Stein *et al* (3) showed that the five-year survival rate was 80% for colorectal cancer patients with low MACC1 expression, but 15% for patients with high MACC1 expression. Previous studies have also demonstrated that c-MET is a prognostic factor for colon cancer, however, Stein *et al* showed that the combination of MACC1 and c-MET expression did not improve the prognosis for five-year survival or metastasis, indicating that MACC1 may serve as an independent prognostic factor. In addition, several studies have also demonstrated the clinical link between MACC1 and tumors. Shimokawa *et al* (13) showed that the expression of MACC1 was significantly higher in recurrent lung adenocarcinomas than in non-recurrent ones, and that patients with positive MACC1 staining had poorer DFS. Shirahata *et al* (14,15) also observed that MACC1 expression in hepatocellular and gastric cancers was significantly higher than in corresponding normal tissues. In addition, MACC1 was found to correlate with vascular invasion and α-fetoprotein levels in hepatocellular carcinoma and with peritoneal dissemination in gastric cancer. In gynecological cancers, studies (16,17) have shown that compared with normal ovarian tissues and benign cancer tissues, ovarian cancer tissues exhibit higher MACC1 expression levels. High MACC1 expression was associated with advanced International Federation of Obstetricians and Gynecologists stage, poor differentiation and lymph node metastasis. Furthermore, the transfection of MACC1-siRNA into OVCAR3 cells was found to significantly reduce invasion and metastasis *in vitro* and *in vivo*. The RNA interference (RNAi) technique (18), which is the most effective antisense technique, is important in the study of gene function and the gene therapy of tumors.

In the present study, RNAi technology was employed to knock down endogenous MACC1 expression and to analyze the effect of MACC1 on the metastatic behavior of ovarian cancer cells. qPCR and western blot analysis confirmed that MACC1-siRNA effectively suppressed the expression of MACC1 in OVCAR3 cells, with inhibition rates of 75.7% at the mRNA level and 55.3% at the protein level. Next, the major malignant characteristics of the ovarian cancer cells, including adhesion, migration, invasiveness and angiogenesis, which are essential steps for the establishment of metastasis, were investigated. Adhesion and CCK-8 assays confirmed that the MACC1-siRNA sequence altered the adhesion and proliferation of the cells. A wound-healing assay also demonstrated the significantly slower migration in the MACC1-silenced group compared with the control groups, which was further corroborated by the Transwell assay. Following the transfection of MACC1-siRNA into the OVCAR3 cells, the invasion and vascularization capacities were significantly decreased. All the results indicated that MACC1 is important for the invasion and migration of ovarian cancer.

Previous studies have found that c-MET encodes the HGF receptor and consists of 21 exons interrupted by 20 introns. When the complete structural organization and promoter characterization of c-MET was analyzed in renal epithelial mIMCD3 cells, Liu (19) identified two positive regulatory elements and one negative regulatory element in the promoter of the 5′-regulatory region, which were located at nucleotide positions −2615 to −1621, −223 to −68, and −1621 to −1093, respectively. Stein *et al* (3) also identified c-MET as one of the targets of MACC1 and reported that MACC1 binds between fragments −223 and −68 of the c-MET promoter, transcriptionally regulating its expression. MACC1 induces HGF/c-MET signaling pathway activation, resulting in enhanced cell motility, invasion and metastasis. c-MET is a metastasis promoter and is overexpressed in a variety of tumors. Increasing evidence has demonstrated a link between c-MET overexpression and increased tumor cell metastasis and invasion. In previous studies (20,21), c-MET overexpression in ovarian carcinoma has been associated with advanced tumor stage, and the knockdown of endogenous c-MET expression using siRNA has been found to greatly reduce the invasive ability of the cells. Attenuated c-MET expression also weakens the invasiveness and metastasis of colon cancer and hepatocellular carcinoma (22,23). As a result, the current study investigated the potential association between c-MET and MACC1 in ovarian cancer. MACC1-siRNA transfected into OVCAR3 cells was found to significantly decrease the levels of c-MET protein compared with the control cells. Furthermore, a luciferase reporter assay confirmed that c-MET is a target of MACC1 in ovarian cancer cells. Consistent with these results, Stein *et al* (9) has also shown that MACC1 can directly bind to the c-MET promoter.

In conclusion, the current study reported that MACC1 downregulation may effectively suppress the malignant biological behavior of ovarian cancer and confirmed that c-MET is a target of MACC1. MACC1 may be important in regulating the tumorigenesis and development of ovarian cancer. In addition, it is indicated that MACC1 inhibition may be a novel pharmaceutical target for inhibiting ovarian cancer metastasis.

## References

[1] Siegel R, Ward E, Brawley O, Jemal A (2011). Cancer statistics, 2011: the impact of eliminating socioeconomic and racial disparities on premature cancer deaths. CA Cancer J Clin.

[2] Chen J, Liu X, Zhang J, Zhao Y (2012). Targeting HMGB1 inhibits ovarian cancer growth and metastasis by lentivirus-mediated RNA interference. J Cell Physiol.

[3] Stein U, Walther W, Arlt F (2009). MACC1, a newly identified key regulator of HGF-MET signaling, predicts colon cancer metastasis. Nat Med.

[4] Shirahata A, Shinmura K, Kitamura Y (2010). MACC1 as a marker for advanced colorectal carcinoma. Anticancer Res.

[5] Qiu J, Huang P, Liu Q (2011). Identification of MACC1 as a novel prognostic marker in hepatocellular carcinoma. J Transl Med.

[6] Chundong G, Uramoto H, Onitsuka T (2011). Molecular diagnosis of MACC1 status in lung adenocarcinoma by immunohistochemical analysis. Anticancer Res.

[7] Tachibana K, Minami Y, Shiba-Ishii A (2012). Abnormality of the hepatocyte growth factor/MET pathway in pulmonary adenocarcinogenesis. Lung Cancer.

[8] You WK, McDonald DM (2008). The hepatocyte growth factor/c-Met signaling pathway as a therapeutic target to inhibit angiogenesis. BMB Rep.

[9] Stein U, Dahlmann M, Walther W (2010). MACC1-more than metastasis? Facts and predictions about a novel gene. J Mol Med.

[10] Qin X, Yan L, Zhao X, Li C, Fu Y (2012). microRNA-21 overexpression contributes to cell proliferation by targeting PTEN in endometrioid endometrial cancer. Oncol Lett.

[11] Ye Q, Yan Z, Liao X (2011). MUC1 induces metastasis in esophageal squamous cell carcinoma by upregulating matrix metalloproteinase 13. Lab Invest.

[12] Wu ZS, Wang CQ, Xiang R (2012). Loss of miR-133a expression associated with poor survival of breast cancer and restoration of miR-133a expression inhibited breast cancer cell growth and invasion. BMC Cancer.

[13] Shimokawa H, Uramoto H, Onitsuka T (2011). Overexpression of MACC1 mRNA in lung adenocarcinoma is associated with postoperative recurrence. J Thorac Cardiovasc Surg.

[14] Shirahata A, Fan W, Sakuraba K (2011). MACC 1 as a marker for vascular invasive hepatocellular carcinoma. Anticancer Res.

[15] Shirahata A, Sakata M, Kitamura Y (2010). MACC 1 as a marker for peritoneal-disseminated gastric carcinoma. Anticancer Res.

[16] Zhang RT, Shi HR, Huang HL (2011). Expressions of MACC1, HGF, and C-met protein in epithelial ovarian cancer and their significance. Nan Fang Yi Ke Da Xue Xue Bao.

[17] Zhang R, Shi H, Chen Z (2011). Effects of metastasis-associated in colon cancer 1 inhibition by small hairpin RNA on ovarian carcinoma OVCAR-3 cells. J Exp Clin Cancer Res.

[18] Chen XQ, Yang S, Kang MQ (2012). Survivin expression in human lung cancer and the influence of its downregulation on the biological behavior of human lung cancer cells. Exp Ther Med.

[19] Liu Y (1998). The human hepatocyte growth factor receptor gene: complete structural organization and promoter characterization. Gene.

[20] Mitra AK, Sawada K, Tiwari P (2011). Ligand-independent activation of c-Met by fibronectin and α(5)β(1)-integrin regulates ovarian cancer invasion and metastasis. Oncogene.

[21] Bu R, Uddin S, Bavi P (2011). HGF/c-Met pathway has a prominent role in mediating antiapoptotic signals through AKT in epithelial ovarian carcinoma. Lab Invest.

[22] Seiden-Long IM, Brown KR, Shih W (2006). Transcriptional targets of hepatocyte growth factor signaling and Ki-ras oncogene activation in colorectal cancer. Oncogene.

[23] You H, Ding W, Dang H, Jiang Y, Rountree CB (2011). c-Met represents a potential therapeutic target for personalized treatment in hepatocellular carcinoma. Hepatology.

